# Measles Epidemics Among Children in Vietnam: Genomic Characterization of Virus Responsible for Measles Outbreak in Ho Chi Minh City, 2014

**DOI:** 10.1016/j.ebiom.2014.10.015

**Published:** 2014-10-29

**Authors:** Van H. Pham, Diem P.H. Nguyet, Khanh N.H. Mai, Khanh H. Truong, Loc V. Huynh, Trang H.T. Pham, Kenji Abe

**Affiliations:** a Center for Molecular Biomedicine, School of Medicine, University of Medicine and Pharmacy in Ho Chi Minh City, Ho Chi Minh City, Vietnam; b Molecular Diagnostics Section, Nam Khoa-Biotek Laboratory, Ho Chi Minh City, Vietnam; c Department of Respiratory Diseases, Children Hospital No. 1, Ho Chi Minh City, Vietnam; d Department of Infectious Diseases, Children Hospital No. 1, Ho Chi Minh City, Vietnam; e Department of Pathology, National Institute of Infectious Diseases, Tokyo, Japan

**Keywords:** Measles, Measles virus, Virus genotype, Vietnam, Southeast Asia

## Abstract

**Background:**

Measles remains poorly controlled in Southeast Asia, including Vietnam.

**Objectives:**

The aim of this study was to characterize genes of virus responsible for a measles outbreak among children in Vietnam in 2014.

**Study design:**

Throat swab samples were collected from 122 pediatric patients with suspected measles. Furthermore, peripheral blood mononuclear cells (PBMCs) from 31 of these cases were also collected. Measles virus (MV) RNA was obtained directly from the clinical specimens, amplified by PCR, and then the N and H genes were sequenced.

**Results:**

MV RNA was detectable in throat swabs from all 122 patients tested. Positive-strand viral RNA, which indicates the intermediate replicative form of MV, was also detected in PBMCs from all 31 cases from whom these cells were collected. One hundred and eighteen strains with the N gene were obtained by RT-PCR and sequenced. Using phylogenetic analysis with measles reference sequences, all of the Vietnamese strains were found to be genotype D8. However, all strains formed a distinct cluster within the genotype D8 group (D8-VNM) suggesting their own lineage. This distinct cluster was supported by a branch with a 99% bootstrap value and 3.3% nucleotide divergence in the N-450 region of the N gene from the D8 reference strain. Notably, all of the D8-VNM variant strains represented unique amino acid sequences consisting of R442, S451 and G452 in the N-450 region of the N gene.

**Conclusions:**

Measles viruses responsible for outbreaks in Southern Vietnam belonged to a genotype D8 variant group which had unique amino acid sequences in the N gene. Our report provides important genomic information about the virus for measles elimination in Southeast Asia.

## Highlights

• Measles viruses responsible for outbreaks among children in Southern Vietnam in 2014, belonged to genotype D8 variant group which had unique amino acid sequences in the N-450 region of the N gene.
• Our report provides important genomic information of the virus for measles elimination in Southeast Asia.

## 1 Introduction

Although measles is now a vaccine-preventable disease, it remains one of the leading causes of death in children, especially in resource-poor regions of the world (Moss and Griffin, 2006, Nandy et al., 2006, Wolfson et al., 2009). Therefore, it is very important to control the measles in order to improve child health worldwide. Measles is a highly contagious respiratory viral disease characterized by the appearance of fever and a rash that can be very serious. Although improvements have been made to the control of measles worldwide by the WHO, large-scale outbreaks have recently been observed, particularly in developing countries including those of Southeast Asia (Simons et al., 2012, Perry et al., 2014).

The measles virus (MV) is a single-stranded, negative-sense RNA virus, belonging to the genus *Morbillivirus*, family *Paramyxoviridae*. It consists of 15,894 nucleotides (nt) encoding six structural proteins. Although MV is thought to be serologically monotypic, high genetic variability in the N and H genes has been known. In particular, the 450 nt C-terminus of the N gene is the target area for MV genotyping recommended by the WHO (Expanded Programme on Immunization (EPI), 1998, Anon., 2005, Anon., 2006, Anon., 2012).

Sniadack et al. reported on large-scale measles epidemics in Vietnam during 2008–2010 (Sniadack et al., 2011). However, detailed molecular-based epidemiologic studies on MV circulating in Vietnam have not yet appeared. Genetic information of the viruses has been used in molecular epidemiologic studies to identify the transmission pathways and the route of infection. Therefore, genetic surveillance of the MV has provided a means to measure the success of the measles control program (Liffick et al., 2001).

Here we report on genomic characterization of a large-scale measles outbreak in Vietnam in 2014. In this study, we focused on the N-450 region to characterize the MV circulating in Vietnam and succeeded in determining that the virus has a unique genomic characterization.

## 2 Materials and Methods

### 2.1 Patient Samples

Throat swab samples were obtained from 122 pediatric patients (aged from 2 months to 14 years old; 46 cases of less than one year of age and 76 cases older than 1 year; 70 boys and 52 girls) with suspected measles having symptoms of severe rash typical of measles, high fever for 2–3 days, cough, coryza, red eyes and Koplik's spot at the Children Hospital No. 1, Ho Chi Minh City, Vietnam that have occurred between February to March 2014. Furthermore, peripheral blood mononuclear cells (PBMCs) from 31 of these cases were also collected. The clinical samples were collected within 8 days after the onset of rash. All clinical samples were stored frozen at − 80 °C until use.

### 2.2 Ethical Approval

An approval for this study was obtained from the Children Hospital No. 1 Research Ethics Board. Fully informed, written consent was obtained from parents or legal guardians of all of the children patients that participated in this study.

### 2.3 Separation of PBMCs

PBMCs were separated by Ficoll–Isopaque (Nacalai Tesque, Kyoto, Japan) density-gradient centrifugation, and washed three times with PBS (pH 7.4) to remove free virus in circulating blood. The cell pellet obtained was resuspended in 1 mL of RPMI-1640 media (Life Technologies, Rockville, MD, USA) containing 10% dimethyl sulfoxide and frozen in liquid nitrogen until use for RNA extraction.

### 2.4 Determination of MV RNA by Real-time PCR

For screening of MV RNA determination, the real-time PCR was used with primers designed from the N gene: 5′-TTATTTGTGGAGTCTCCAGGTC-3′ (MV342F; sense, nt 342–363), 5′-CCTCATCCTCCATGTTGGTAC-3′ (MV486R; antisense, nt 486–466), and FAM-5′-AGAGGATCACCGATGACCCTGACG-3′-BHQ1 (labeled probe; nt 373–396). Nucleotide position is based on measles virus vaccine strain Changchun-47 (accession #: FJ416068).

Total RNA was extracted from throat swab and PBMC samples using the magnetic bead-based genomic RNA/DNA purification method (^NK^DNARNAprep-MAGBEAD kit, Nam Khoa-Biotek, Ho Chi Minh, Vietnam). Viral cDNA was synthesized and amplified by the same method as reported previously (Pham et al., 2013). The sensitivity of this assay was 100 copies of MV/mL.

### 2.5 Detection of MV RNA by RT-PCR and Sequence

The sequences of primers used for RT-PCR to detect MV RNA are as follows: Tag-MV1108F: 5′-gtaaaacgacggccagtGCTATGCCATGGGAGTAGGAGTGG-3′ (sense, nt 1108–1131; tag sequences of M13 universal primer are shown by lowercase letters) and Tag-MV1697R: 5′-tatttaggtgacactatagGGCCTCTCGCACCTAGTCTAG-3′ (antisense, nt 1697–1677; tag sequences of SP6 universal primer are shown by lowercase letters) which can yield a 590-bp amplicon in the N gene containing the WHO-recommended sequence window (N-450 region; nt 1233–1682). Furthermore, an entire sequence of the H gene was also amplified by the nested RT-PCR with the primer combination of MV6963F: 5′-GTGTCTTGGAGGRTTGATAGGGA-3′ (sense, nt 6963–6985) and MV9388R: 5′-CGGTGCTTGATGTTCTGACAC-3′ (antisense, nt 9388–9368) for the outer primer pairs (2426 bases), and MV7081F: 5′-ACATCAAAATCYTATGTAAGGTC-3′ (sense, nt 7081–7103) and MV9292R: 5′-ATCGGGCTATCTAGGTGAAC-3′ (antisense, nt 9292–9273) for the inner primer pairs (2212 bp).

Viral cDNA obtained for the real-time PCR was amplified using QIAGEN Multiplex PCR Kit (Qiagen Inc., Chatsworth, CA, USA). For N gene amplification, PCR conditions included pre-incubation at 95 °C for 15 min, followed by 40 cycles consisting of 95 °C for 30 s, 65 °C for 30 s and 72 °C for 1 min. For H gene amplification, MV cDNA was amplified by the nested PCR with the following conditions: 35 cycles consisting of 95 °C for 30 s, 50 °C for 30 s and 72 °C for 2 min 30 s for the 1st PCR and 95 °C for 30 s, 55 °C for 30 s and 72 °C for 2 min 30 s for the 2nd PCR. Obtained amplicons were analyzed and subjected to direct sequencing by the same method as reported previously (Pham et al., 2013).

### 2.6 Detection of Positive-strand MV-RNA in PBMCs

To detect positive-strand of MV-RNA, viral cDNA was synthesized with MV-specific antisense primer (MV486R) using iScript reverse transcriptase with the following condition: 25 °C for 5 min, 42 °C for 30 min and 95 °C for 5 min. Obtained viral cDNA was determined by the real-time PCR with the same condition as described above.

### 2.7 Characterization of MV Gene by Phylogenetic Analysis

For phylogenetic analysis, obtained nucleotide sequences were multiple aligned with CLUSTAL W, version 1.81 as reported previously (Pham et al., 2013). The distance matrix of the nucleotide substitutions among each sequence was estimated by the eight-parameter method and phylogenetic trees were constructed by the neighbor-joining method from the matrix. These procedures were computed with Phylo_win, version 1.2 on a DEC alpha 2000 server, and the trees were drawn with TreeView, version 1.5.2. To confirm the reliability of the pairwise comparison and phylogenetic tree analysis, bootstrap resampling and reconstruction were carried out 1000 times. Bootstrap values greater than 60% were considered supportive of the observed groupings. In addition to our sequences, 28 reference strains recommended by the WHO and 142 strains of genotype D8 obtained from database including Measles Nucleotide Surveillance and GenBank were used as reference strains of known genotypes.

### 2.8 Accession Numbers Submitted to Database

Nucleotide sequence data of MV strains from 118 Vietnamese patients are available in the DDBJ/EMBL/GenBank databases under the accession numbers AB928085 to AB928202AB928085AB928086AB928087AB928088AB928089AB928090AB928091AB928092AB928093AB928094AB928095AB928096AB928097AB928098AB928099AB928100AB928101AB928102AB928103AB928104AB928105AB928106AB928107AB928108AB928109AB928110AB928111AB928112AB928113AB928114AB928115AB928116AB928117AB928118AB928119AB928120AB928121AB928122AB928123AB928124AB928125AB928126AB928127AB928128AB928129AB928130AB928131AB928132AB928133AB928134AB928135AB928136AB928137AB928138AB928139AB928140AB928141AB928142AB928143AB928144AB928145AB928146AB928147AB928148AB928149AB928150AB928151AB928152AB928153AB928154AB928155AB928156AB928157AB928158AB928159AB928160AB928161AB928162AB928163AB928164AB928165AB928166AB928167AB928168AB928169AB928170AB928171AB928172AB928173AB928174AB928175AB928176AB928177AB928178AB928179AB928180AB928181AB928182AB928183AB928184AB928185AB928186AB928187AB928188AB928189AB928190AB928191AB928192AB928193AB928194AB928195AB928196AB928197AB928198AB928199AB928200AB928201AB928202 for N gene and AB968375 to AB968382AB968375AB968376AB968377AB968378AB968379AB968380AB968381AB968382 for H gene.

## 3 Results

### 3.1 Clinical Findings

All pediatric patients with measles were recovered in the course of the transient. Clinical findings of 122 childhood measles patients were as follows; severe rash typical of measles (122/100%), high fever (122/100%), cough (121/99%), coryza (121/99%), red eyes (112/92%), pneumonia (25/20.5%), Koplik's spot (17/14%), and diarrhea (4/3.3%), but none suffered from encephalitis, otitis media, corneal ulceration, croup and mouth ulcer. Among 76 measles patients who were older than 1 year, measles vaccination status was documented in 17 cases (22.4%) who received one injection and one case who received two injections (1.3%); all remaining cases (76.3%) did not receive a vaccination.

### 3.2 Detection of MV RNA and Genomic Characterization of the Virus

By screening with real-time PCR, MV RNA was detectable in throat swab samples from all 122 patients with clinically suspected measles.

**Fig. 1:**
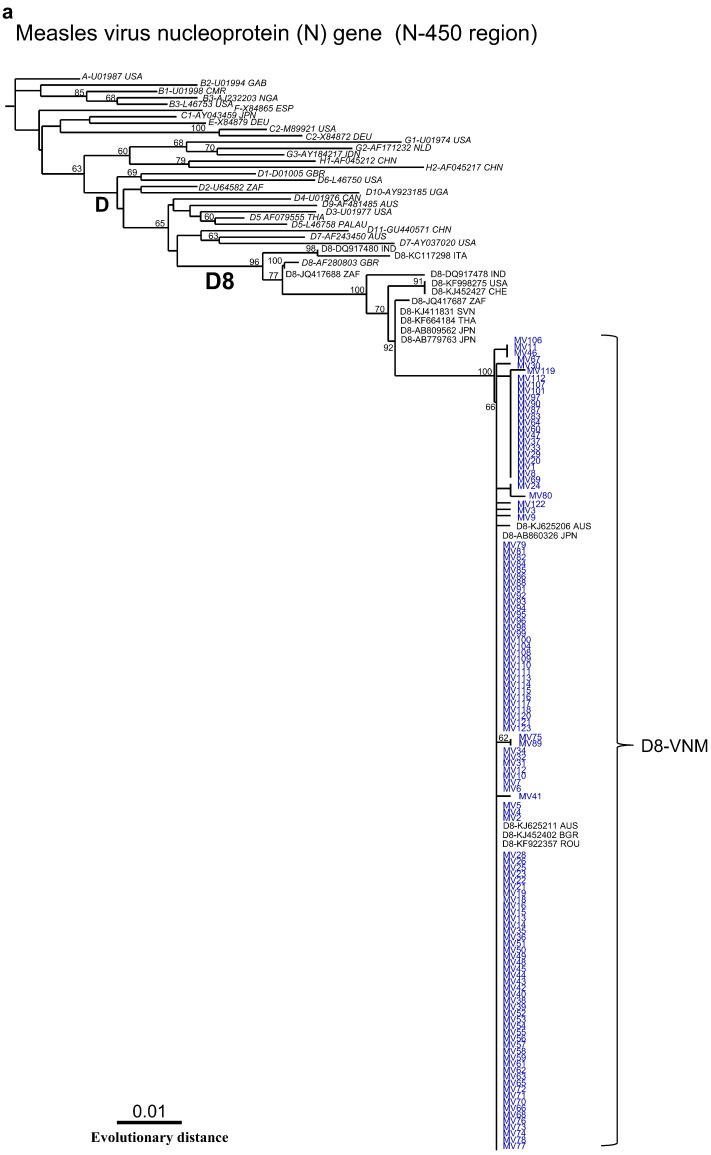

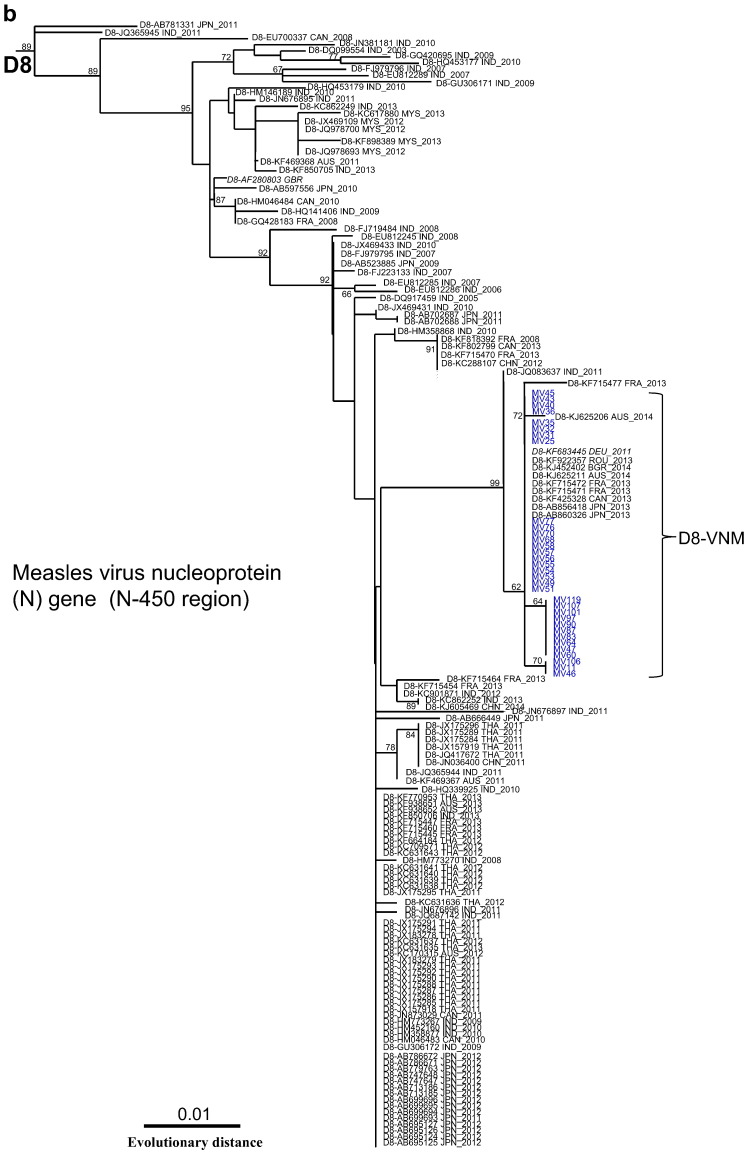
Phylogenetic tree generated by neighbor-joining analysis of genetic distances in the N-450 region of the N gene based on WHO-recommended sequence window. Tree was constructed with 28 reference strains from all genotypes recommended by WHO (1a) and 142 strains of genotype D8 obtained from database (1b). Because of limited space, the number of strains of D8-VNM isolates has been reduced in Fig. 1b. Vietnamese strains identified in this study are indicated in blue. WHO reference strains are indicated in italics. Bootstrap values of > 60% are shown at the branch nodes. (For interpretation of the references to color in this figure legend, the reader is referred to the web version of this article.)

One hundred and eighteen strains in the N gene were obtained by RT-PCR and sequenced. The Vietnamese MVs recovered were all closely related at the nucleotide level with 0–0.4% divergence in the N-450 region. The mean divergence within all Vietnamese viral sequences was 0.3%, but there was 3.3–11.3% nucleotide divergence over the N-450 region compared with WHO reference strains (Fig. 1).

Using phylogenetic analysis with measles reference sequences, all of the Vietnamese strains were clustered within clade D and belonged to the genotype D8 group (Fig. 1a). However, interestingly, all of the Vietnamese D8 strains formed an independent cluster within the same genotype D8 group, suggesting their own lineage with specific genetic variations (D8-VNM variants) (Fig. 1b). This finding was supported by a branch with the high bootstrap value of 99% and 3.3% nucleotide divergence in the N-450 region from the WHO/D8 reference strain, MVi/Mancherster.UNK/30.94 (accession #: AF280803) (Fig. 1b). The WHO lineage strain, MVs/Frankfurt Main.DEU/17.11 (accession #: KF683445), and other database-derived strains isolated from various countries including France, Canada, Australia, India and Japan are also grouped in the same cluster of D8-VNM (Fig. 1b). All strains that belonged to the D8-VNM variant group have been isolated from 2011 through 2014. Among these, Indian strain MVs/Pune.Ind/03.11/O.F (accession #: JQ083637) was located genetically in the most distant position in the phylogenetic tree, suggesting that it is probably the closest to the origin of the virus of the D8-VNM variant group at the moment (Fig. 1b).

**Fig. 2:**
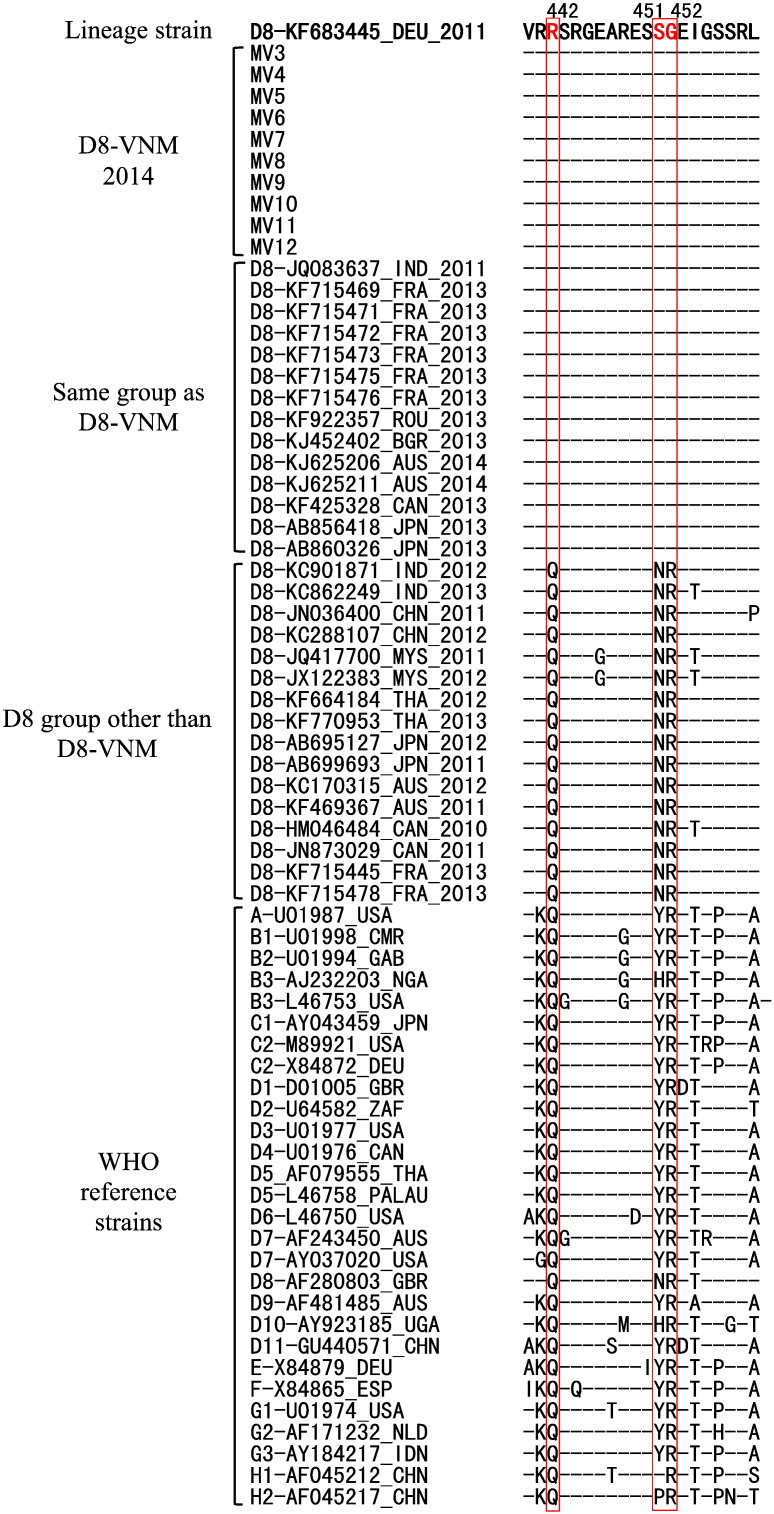
Multiple alignment of amino acid sequences in the N-450 region of the N gene shows unique amino acid sequences consisting of R442, S451 and G452 among D8-VNM group.

Furthermore, notably, when amino acid sequences in the N-450 region were compared with other D8 and other genotype strains, all strains grouped into D8-VNM had unique amino acid sequences consisting of R442, S451 and G452 in the N gene (Fig. 2).

**Fig. 3:**
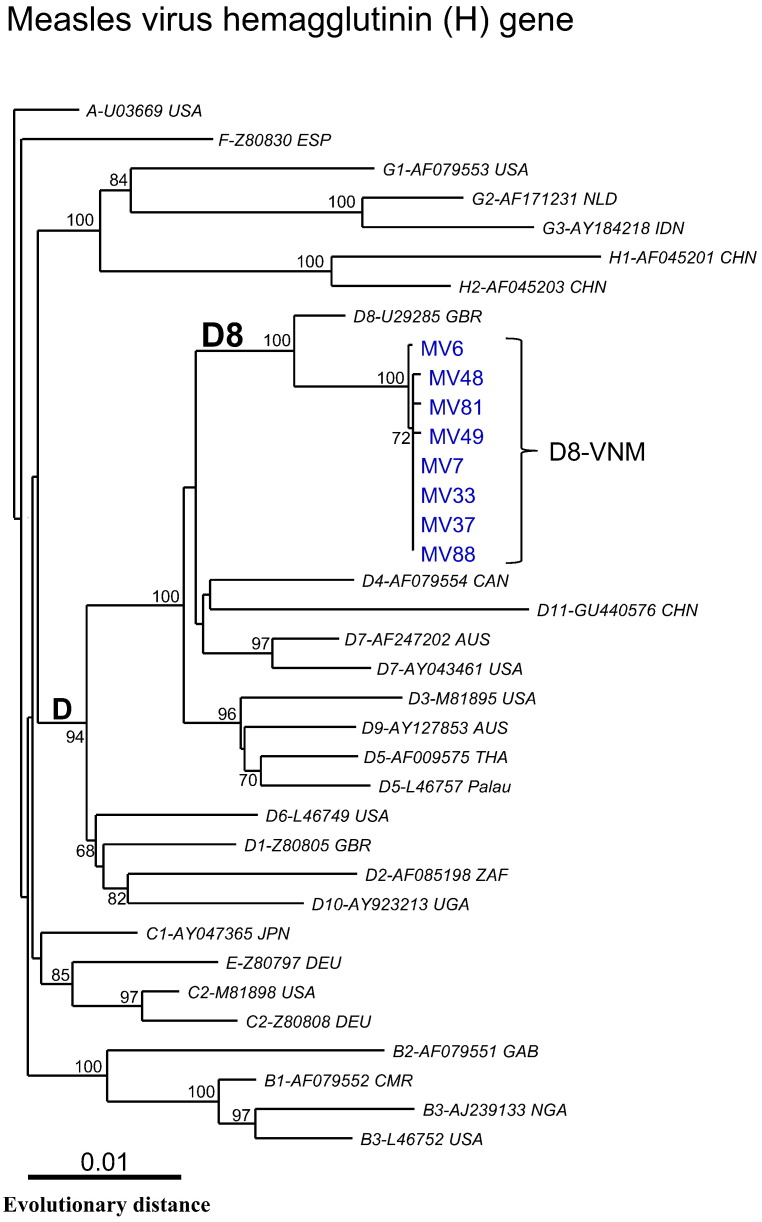
Phylogenetic tree generated by neighbor-joining analysis of genetic distances in the entire H gene. Tree was constructed with 28 reference strains from all genotypes recommended by the WHO. Vietnamese strains identified in this study are indicated in blue. WHO reference strains are indicated in italics. Bootstrap values of > 60% are shown at the branch nodes.

In addition, in order to further characterize the D8-VNM viruses, the complete coding region of the H gene from 8 throat swab samples with high viral copies was sequenced. As a result, the Vietnamese MVs were found to be closely related at the nucleotide level, showing 0.05–0.1% divergence within their own group and 1.1% nucleotide divergence when compared with the WHO/D8 reference strain (accession #: U29285) in the complete coding region of the H gene. Phylogenetic analysis using the complete H gene showed that all 8 Vietnamese strains belonged to genotype D8 as shown by the N gene (Fig. 3).

### 3.3 Detection of Positive-strand MV RNA in PBMCs

The positive-strand MV RNA, which is an intermediate replicative form of negative-strand RNA viruses, was detectable in all of the PBMCs obtained from 31 Vietnamese children with measles.

## 4 Discussion

Although measles is now a vaccine-preventable disease, measles remains one of the most important causes of child morbidity and mortality worldwide with the greatest burden in the youngest children (Moss and Griffin, 2006, Nandy et al., 2006, Wolfson et al., 2009). Vaccination programs have dramatically reduced the incidence of measles in developed countries, however, vaccination coverage differs widely. Globally, measles mortality decreased by 78% in the period from 2000 to 2008 (Simons et al., 2012, O'Connor et al., 2011). In Southeast Asian countries, the estimated measles mortality has decreased dramatically and all countries except India have achieved the 90% mortality reduction target. In fact, routine measles-containing vaccine coverage in this region increased from 63% in 2000 to 75% in 2008. However, in 2008, about 9 million children born in this region were not vaccinated against measles (World Health Organization, 2010). As a result, measles still remains poorly controlled in many countries in Southeast Asia.

Vietnam is a country with a high endemic level of measles and rubella, although the WHO has reported that the measles-containing vaccine coverage rate is more than 90% in this country (World Health Organization, 2010). The two-dose strategy of measles/rubella immunization has been implemented in Vietnam at the ages of 9–11 months and 18 months since 2006, but large-scale measles outbreaks have still occurred among children. Sniadack et al. reported on the epidemiological features of large-scale measles outbreaks in this country during 2008–2010 (Sniadack et al., 2011). However, until now, no large-scale survey on measles epidemics to characterize the viral genome circulating in Vietnam has yet been reported. From the end of 2013 through 2014, large outbreaks of measles among children have recurred in this country. Unfortunately, in our study presented here, we were not able to obtain detailed epidemiological information including the epidemic curve and background of the 2014 measles outbreaks in Ho Chi Minh City and neighboring provinces since the infectious disease surveillance system in Vietnam is not yet functional. During the same period, a measles outbreak also occurred in the Hanoi area which is in the Northern part of Vietnam, however, no detailed information, including the viral genotypes, is available. Very recently, we learned that genotype H1 of MV has been detected in a few cases from the 2014 outbreaks in Hanoi area (Anon., 2014). If so, different viral strains could be responsible for the 2014 measles outbreaks in the Southern and Northern parts of Vietnam. This reason might be due to geographical factors. Hanoi is located near the border of China and genotype H1 of MV is mainly circulating in China; consequently, viral strains isolated in the Hanoi area seem to be often linked to Chinese strains.

The use of the molecular epidemiologic approach has contributed to understanding of the worldwide genetic diversity and transmission routes of pathogens and is considered important for supporting activities aimed at control and elimination of disease. Based on the C-terminal sequence of the N gene, the WHO currently recognizes 8 clades designated as A–H and within these clades there are 24 recognized genotypes based on sequence variation in the 450-nt of the N gene (Anon., 2012). Genotypic distribution of MV has been reported from many different regions, but there have been very few reports from Southeast Asia. A recent epidemiological study from Thailand reported that three different viral genotypes, D5, D9 and G2, were identified from 1998 to 2008 and D9 was the most frequently detected in 2008 (Pattamadilok et al., 2012).

In this study, we used throat swabs obtained from suspected measles patients to detect the viral RNA and obtained a very high detection rate (100%) of MV RNA by RT-PCR. Our results reported here could be useful in efforts to control measles in Vietnam and adjacent neighboring countries. In our study based on molecular-based epidemiology of measles, we were able to confirm that the genotype D8 viruses are responsible for the measles outbreak in Vietnam in 2014. The genotype D8 virus was previously known to be prevalent in India and Europe, but has been relatively rare in Asia (Rota et al., 2011). Interestingly, although the phylogenetic analysis revealed that the Vietnamese MVs belonged to the genotype D8 clade, they formed an independent cluster within the same genotype D8 group and were located furthest away among the D8 group, suggesting their own lineage in the genomic variation of the Vietnamese viruses. Our recent study on rubella epidemics in Vietnam also showed similar findings that most of the Vietnamese virus strains responsible for rubella outbreaks formed an independent cluster within the same genotype 2B group (Pham et al., 2013). Notably, all Vietnamese measles viruses identified in this study had a unique amino acid sequence in at least 3 different sites in the N protein. The viral N protein could play an important role in viral RNA synthesis since it forms a helical nucleocapsid around the genomic RNA to form the ribonucleocapsid (Longhi et al., 2003, Griffin et al., 2012). The relationship between the virus with such unique amino acid sequences, the efficacy of the measles vaccine and the viral pathogenicity in the clinical outcome is awaited with great interest.

In this study, positive-strand MV sequence was detected in all PBMC samples collected within 8 days after the onset of rash. MV infection of mononuclear cells leads to an immunocompromised condition that often results in a fatal form of the disease (Sullivan et al., 1975, Griffin, 2010). With the recent recognition that MV RNA can persist in PBMCs for months after clearance of the infectious virus (Pan et al., 2005, Riddell et al., 2007), detailed studies are needed to determine the state of MV–lymphocyte interactions and their relationship with pathogenesis of a fatal course or certain autoimmune diseases.

In conclusion, the present study clarified that MVs responsible for the 2014 measles outbreaks in Southern Vietnam belonged to a genotype D8 variant group, which is characterized by having unique amino acid sequences in the N gene. Our results indicate that the establishment of measles prevention in this area is an urgent task in order to improve child health in the Southeast Asian continent.

## Funding

None.

## Author Contributions

Designed the study: VHP, DPHN, and KA.

Collected the patient's samples: KNHM, DPHN, and KHT.

Analyzed the clinical data: KNHM.

Performed the experiments and the data collection: LVH, THTP and VHP.

Performed the literature search, the comprehensive final data analysis and the data interpretation: KA.

Wrote the paper: KA.

## Competing Interests

None.

## References

[bb0005] Anon. (2005). New genotype of measles virus and update on global distribution of measles genotypes. Wkly. Epidemiol. Rec..

[bb0010] Anon. (2006). Global distribution of measles and rubella genotypes — update. Wkly. Epidemiol. Rec..

[bb0015] Anon. (2012). Measles virus nomenclature update: 2012. Wkly. Epidemiol. Rec..

[bb0020] Anon. (2014). WHO Western Pacific Region. Measles-Rubella Bull..

[bb0025] Expanded Programme on Immunization (EPI) (1998). Standardization of the nomenclature for describing the genetic characteristics of wild-type measles viruses. Wkly. Epidemiol. Rec..

[bb0030] Griffin D.E. (2010). Measles virus-induced suppression of immune responses. Immunol. Rev..

[bb0035] Griffin D.E., Lin W.H., Pan C.H. (2012). Measles virus, immune control and persistence. FEMS Microbiol. Rev..

[bb0040] Liffick S.L., Thoung T.N., Xu W., Li Y., Phoung L.H., Bellini W.J. (2001). Genetic characterization of contemporary wild-type measles viruses from Vietnam and the People's Republic of China: identification of two genotypes within clade H. Virus Res..

[bb0045] Longhi S., Receveur-Bréchot V., Karlin D., Johansson K., Darbon H., Bhella D. (2003). The C-terminal domain of the measles virus nucleoprotein is intrinsically disordered and folds upon binding to the C-terminal moiety of the phosphoprotein. J. Biol. Chem..

[bb0050] Moss W.J., Griffin D.E. (2006). Global measles elimination. Nat. Rev. Microbiol..

[bb0055] Nandy R., Handzel T., Zaneidou M., Biey J., Coddy R.Z., Perry R. (2006). Case-fatality rate during a measles outbreak in eastern Niger in 2003. Clin. Infect. Dis..

[bb0060] O'Connor P.M., Liyanage J.B., Mach O., Anand A., Ramamurty N., Balakrishnan M.R. (2011). South-East Asia Regional update on measles mortality reduction and elimination, 2003–2008. J. Infect. Dis..

[bb0065] Pan C.H., Valsamakis A., Colella T., Nair N., Adams R.J., Polack F.P. (2005). Inaugural Article: modulation of disease, T cell responses, and measles virus clearance in monkeys vaccinated with H-encoding alphavirus replicon particles. Proc. Natl. Acad. Sci. U. S. A..

[bb0070] Pattamadilok S., Incomserb P., Primsirikunawut A., Lukebua A., Rota P.A., Sawanpanyalert P. (2012). Genetic characterization of measles viruses that circulated in Thailand from 1998 to 2008. J. Med. Virol..

[bb0075] Perry R.T., Gacic-Dobo M., Dabbagh A., Mulders M.N., Strebel P.M., Okwo-Bele J.M. (2014). Global control and regional elimination of measles, 2000–2012. MMWR Morb. Mortal. Wkly. Rep..

[bb0080] Pham V.H., Nguyen T.V., Nguyen T.T., Dang L.D., Hoang N.H., Nguyen T.V. (2013). Rubella epidemic in Vietnam: characteristic of rubella virus genes from pregnant women and their fetuses/newborns with congenital rubella syndrome. J. Clin. Virol..

[bb0085] Riddell M.A., Moss W.J., Hauer D., Monze M., Griffin D.E. (2007). Slow clearance of measles virus RNA after acute infection. J. Clin. Virol..

[bb0090] Rota P.A., Brown K., Mankertz A., Santibanez S., Shulga S., Muller C.P. (2011). Global distribution of measles genotypes and measles molecular epidemiology. J. Infect. Dis..

[bb0095] Simons E., Ferrari M., Fricks J., Wannemuehler K., Anand A., Burton A. (2012). Assessment of the 2010 global measles mortality reduction goal: results from a model of surveillance data. Lancet.

[bb0100] Sniadack D.H., Mendoza-Aldana J., Huyen D.T., Van T.T., Cuong N.V., Olive J.M. (2011). Epidemiology of a measles epidemic in Vietnam 2008–2010. J. Infect. Dis..

[bb0105] Sullivan J.L., Barry D.W., Lucas S.J., Albrecht P. (1975). Measles infection of human mononuclear cells, I. Acute infection of peripheral blood lymphocytes and monocytes. J. Exp. Med..

[bb0110] Wolfson L.J., Grais R.F., Luquero F.J., Birmingham M.E., Strebel P.M. (2009). Estimates of measles case fatality ratios: a comprehensive review of community-based studies. Int. J. Epidemiol..

[bb0115] World Health Organization (2010). WHO vaccine preventable disease: monitoring system 2009 global summary. http://www.who.int/immunization/documents/who_ivb_2009/en/index.html.

